# Hemodynamic differences determining rupture and non-rupture in middle cerebral aneurysms after growth

**DOI:** 10.1371/journal.pone.0307495

**Published:** 2024-08-22

**Authors:** Takayuki Nishiwaki, Taichi Ikedo, Yuji Kushi, Koji Shimonaga, Hiroki Kobayashi, Takaaki Itazu, Ryotaro Otsuka, Jota Tega, Eika Hamano, Hirotoshi Imamura, Hisae Mori, Masanori Nakamura, Takayuki Kato, Shinichi Shirakami, Koji Iihara, Toru Iwama, Hiroharu Kataoka

**Affiliations:** 1 Department of Neurosurgery, National Cerebral and Cardiovascular Center, Suita, Osaka, Japan; 2 Department of Neurosurgery, Daiyukai Hospital, Ichinomiya, Aichi, Japan; 3 Department of Neurosurgery, Gifu University School of Medicine, Gifu, Gifu, Japan; 4 Department of Neurosurgery, Kyoto University, Kyoto, Kyoto, Japan; 5 Department of Mechanical Engineering, Nagoya Institute of Technology, Nagoya, Nagoya, Japan; Coventry University, UNITED KINGDOM OF GREAT BRITAIN AND NORTHERN IRELAND

## Abstract

**Background and purpose:**

Intracranial aneurysm growth is a significant risk factor for rupture; however, a few aneurysms remain unruptured for long periods, even after growth. Here, we identified hemodynamic features associated with aneurysmal rupture after growth.

**Materials and methods:**

We analyzed nine middle cerebral artery aneurysms that grew during the follow-up period using computational fluid dynamics analysis. Growth patterns of the middle cerebral artery aneurysms were divided into homothetic growth (Type 1), de novo bleb formation (Type 2), and bleb enlargement (Type 3). Hemodynamic parameters of the four ruptured aneurysms after growth were compared with those of the five unruptured aneurysms.

**Results:**

Among nine aneurysms (78%), seven were Type 1, one was Type 2, and one was Type 3. Three (43%) Type 1 aneurysms ruptured after growth. Maximum oscillatory shear index after aneurysmal growth was significantly higher in ruptured Type 1 cases than in unruptured Type 1 cases (ruptured vs. unruptured: 0.455 ± 0.007 vs. 0.319 ± 0.042, p = 0.003). In Type 1 cases, a newly emerged high-oscillatory shear index area was frequently associated with rupture, indicating a rupture point. Aneurysm growth was observed in the direction of the high-pressure difference area before enlargement. In Types 2 and 3 aneurysms, the maximum oscillatory shear index decreased slightly, however, the pressure difference values remain unchanged. In Type 3 aneruysm, the maximum OSI and PD values remained unchanged.

**Conclusions:**

This study suggests that hemodynamic variations and growth pattern changes are crucial in rupture risk determination using computational fluid dynamics analysis. High-pressure difference areas may predict aneurysm enlargement direction. Additionally, high maximum oscillatory shear index values after enlargement in cases with homothetic growth patterns were potential rupture risk factors.

## Introduction

The risk of intracranial aneurysm (IA) rupture is related to various factors such as size, location, shape, and hemodynamics [1, 2]. Among these risk factors, IA growth is a strong predictor of rupture and subsequent subarachnoid hemorrhage (SAH) [3]. The annual rupture risk of growing unruptured IA is 4.3%, which is higher than the average rupture rate of 0.95% for unruptured IAs [1, 3]. Therefore, surgical intervention is recommended for most enlarged IAs, although the perioperative complications of surgical intervention remain non-negligible. However, many enlarged aneurysms do not rupture for long periods, suggesting that aneurysmal walls may re-stabilize even after aneurysm growth. Considering that the annual rates of aneurysmal growth and rupture are 3.7% and 0.95% [1, 4], respectively, most aneurysms may be managed with conservative treatments. Knowledge of discriminating unstable aneurysms from restabilized aneurysms after growth may contribute to the development of adequate indications for surgical intervention for IAs after growth.

Computational fluid dynamics (CFD) analysis is a useful tool for studying the hemodynamic characteristics of IA rupture [5–8]. However, CFD analysis in most studies has been limited to a single time point, which does not allow for the investigation of dynamic changes in aneurysmal morphology and hemodynamic conditions over time. As the shape of IAs often changes before and after rupture, utilizing only post-rupture images may be a critical limitation for the risk assessment of aneurysmal rupture.

In the present study, we identified the potential factors related to post-growth aneurysmal rupture using CFD analysis in pre- and post-IA growth images, with or without IA rupture. This novel study may contribute to distinguishing dangerous aneurysms prone to rupture from stable aneurysms and developing a new indication for the management of unruptured IAs.

## Materials and methods

### Patients’ data collection

Retrospective data were collected from a database of IAs at our institution as of October 1^st^, 2021. We had access to information that could identify individual patients during or after data collection. We extracted the records of 725 patients diagnosed with unruptured IAs with a follow-up of more than 1 year. Among them, we identified 150 patients with aneurysm growth.

**Fig 1:**
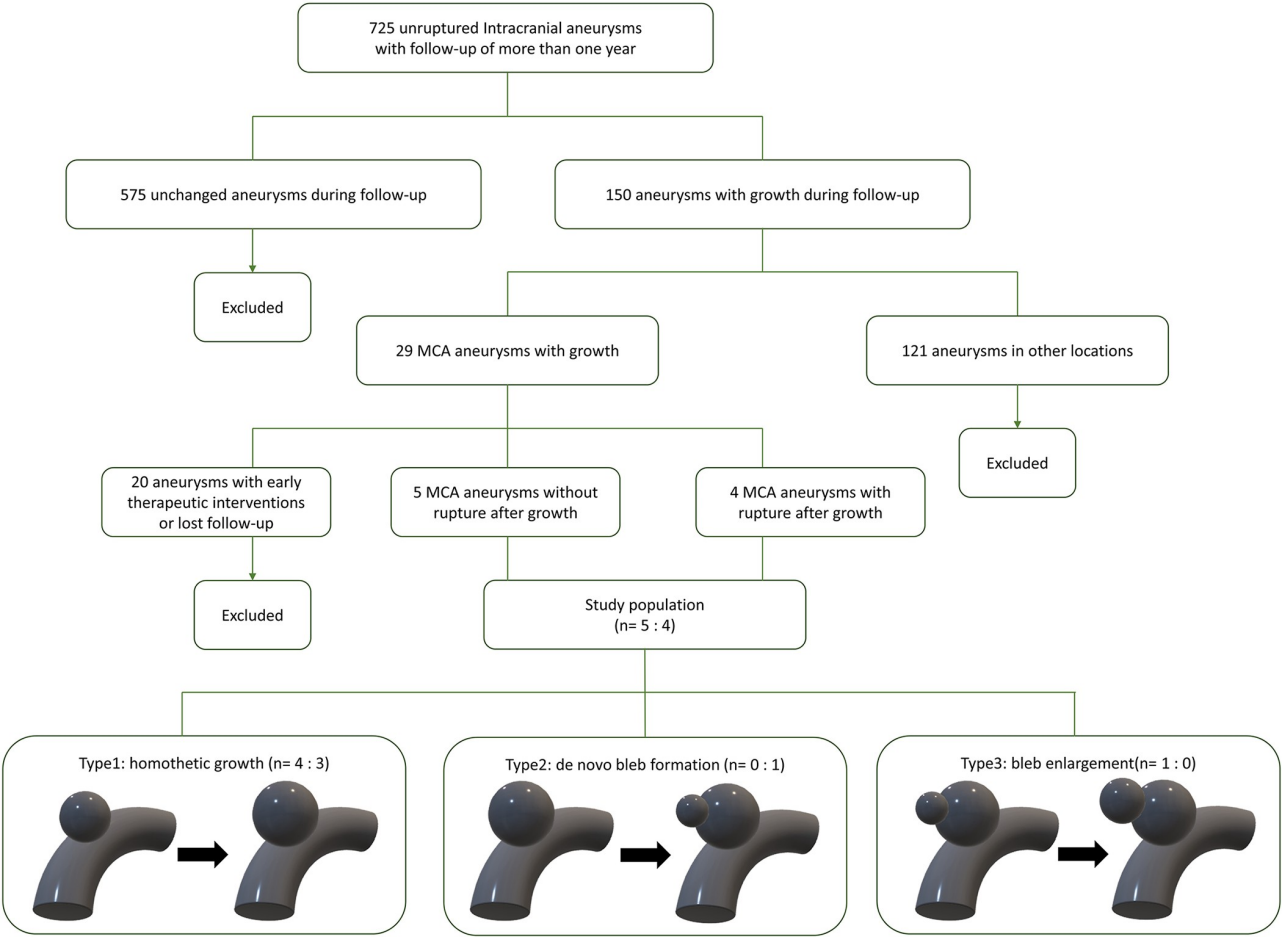
Flow chart of patient population and type of growth patterns. Of the 725 unruptured intracranial aneurysms with a follow-up of more than 1 year, nine MCA aneurysms are included in the present study.

Twenty-nine MCA aneurysms were detected in 150 cases. Among them, nine aneurysms in eight patients without therapeutic intervention after aneurysm growth were included in the present study. One in eight cases had bilateral MCA aneurysms. The remaining 20 aneurysms were excluded because of early therapeutic interventions after growth or loss of follow-up within 1 year. Of the nine aneurysms, four aneurysms in four patients ruptured, whereas five aneurysms in four patients remained unruptured for at least 3 years (up to 6 years) without rupture after growth (Fig 1).

This study was conducted according to the principles expressed in the Declaration of Helsinki (1964) and was approved by the Institutional Review Board of the National Cerebral and Cardiovascular Center. Informed consent was obtained in writing for the use of detailed patient data.

### Imaging and reconstruction for 3D model

The Magnetic Resonance Angiography (MRA) used in this study was performed using the time-of-flight (TOF) method at 3T MRI (*Siemens Healthcare*, *Erlangen*, *Germany*, *or GE Healthcare*, *Chicago*, *IL*). MRAs in the same case were performed using the same MRI model, and the scanning parameters were as follows. Simens MAGNETOM Prisma 3.0T: repetition time = 23 ms, echo time = 3.69 ms, field of view-Read = 200 mm, field of view-Phase = 90.6%, flip angle = 18°, slice thickness = 0.6 mm, matrix = 244–384, voxel = 0.3 × 0.3 × 0.6 mm, the acquisition time was 5.1 minutes; GE SIGNA Premier 3.0T: repetition time = 23 ms, echo time = 3.4–8.7 ms, field of view-Read = 200 mm, field of view-Phase = 94%, flip angle = 18°, slice thickness = 0.3 mm, matrix = 256–384, voxel = 0.5 × 0.8 × 0.6 mm, the acquisition time was 5.5 minutes. Then, the TOF images were reconstructed in 3D in each MRI for the assessment of aneurysm size and shape. Moreover, the data was converted to DICOM in order to be input into the 3D model creation software. The stereolithography (STL) data for the 3D model was created by *Expert INTAGE (Cybernet Systems Co*., *Ltd*., *Tokyo*, *Japan)*. In this software, the threshold was adjusted so that the aneurysm size matched that measured by MRA, and smoothing was also performed.

### Evaluation of aneurysm growth

**Fig 2:**
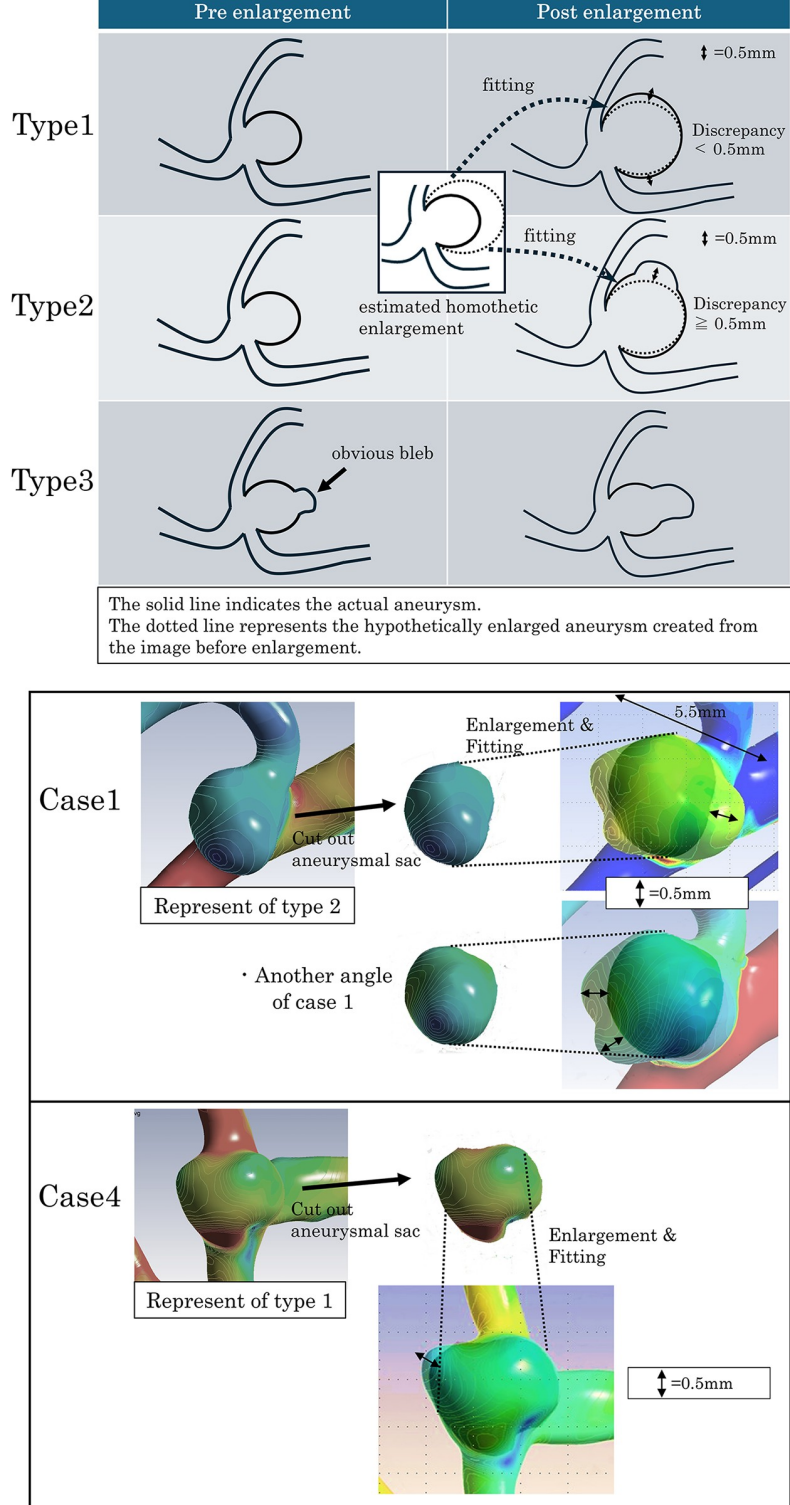
Classification of growth patterns with representative cases.

Aneurysm growth was defined as an enlargement ≥1 mm in at least one direction on the MRA during follow-up and categorized into three types of growth patterns based on the aneurysm shape after growth. The three-type classification was conducted using the following steps with 3D images created from the MRA. Step 1: Cut out only the aneurysmal sac from the image before enlargement and simply enlarge this part to the estimated homothetic shape until the width or height matches the aneurysmal sac after enlargement. Step 2: Superimpose the estimated homothetic enlarged aneurysm onto the post-enlargement image to confirm the discrepant area. Step 3: Measure the length of the discrepant area, and if there is a difference less than 0.5 mm, it should be judged as diffuse enlargement without shape changes, that is, type 1 (homothetic enlargement). If there is a difference of 0.5 mm or more, indicating a suspicion of bleb formation, it should be classified as type 2 (de novo bleb formation). Finally, cases where blebs are obvious in the image before enlargement, and those in which bleb size enlargement is observed, should be defined as type 3 (bleb enlargement, Fig 2).

### CFD simulation

MRA scans obtained at the initial diagnosis and after aneurysm growth were used for hemodynamic simulations. The STL data created with the *Expert INTAGE (Cybernet Systems Co*.*)* was input into *Ansys SpaceClaim (Ansys*, *Inc*., *Berkeley*, *CA)* to create geometry data of blood vessels, and those with MCA aneurysms were extracted from the internal carotid artery (C5 segment) to the M2 segment. The blood flow was set as a Newtonian fluid model with a density of 1,100 kg/m^3^ and viscosity of 0.0036 N⋅s/m^2^. The inlet boundary conditions were set as pulsatile laminar flow, which assumed the typical volumetric rate of flow waveforms in 17 normal volunteers (16 male, 1 female) [9]. The outlet boundary conditions were set to zero pressure. The blood vessel walls were modeled as rigid under a no-slip condition, assuming zero velocity. The mesh structure was consisted of tetrahedral elements (Representative size: 0.2mm) and a prism mesh with five layers (0.05mm height with 1.2 expansion) on the wall surface, which was generated using *Ansys Meshing (Ansys*, *Inc*.*)*. This meshing set the number of elements to be about 15 to 20 in the diameter of the proximal large vessel. The inlet and outlet vessels were extended by a distance equivalent to 10 times their diameter while maintaining the shape of each end face. Calculation of the incompressible Navier–Stokes equations under pulsatile blood flow conditions was performed using *Ansys CFD (Ansys*, *Inc*.*)*.

### Data analysis and quantification

We calculated the following previously reported hemodynamic parameters related to the rupture of IAs: wall shear stress (WSS), wall shear stress gradient (WSSG), oscillatory shear index (OSI), and pressure difference (PD). WSS is a mechanical force acting on the endothelial cells of the blood vessel wall owing to friction between the blood flow and the vessel wall. As WSS was calculated instantaneously in each cardiac cycle, we used time-averaged WSS for evaluation in contour images. WSSG is defined as the spatial derivative of the WSS vector magnitude in the flow direction [10] and represents the changes in WSS along the streamwise distance. OSI measures the spatial and temporal variations in the WSS direction, indicates the magnitude of WSS changes, and illustrates the tangential oscillation of force over one cardiac cycle. PD describes the difference in blood pressure between the target part and the inlet side [11] and represents the pressure elevation degree at the aneurysm wall. It is calculated by subtracting the average pressure from the maximum pressure and dividing it by the dynamic pressure at the aneurysm inlet side for normalization.

As the above parameters were calculated from any part of the vessel wall, the aneurysm neck plane was defined to separate the aneurysm wall and parent vessel for spatial quantification. Additionally, a plane perpendicular to the vessels was placed 2 mm away from the aneurysm such that the values at the inlet and outlet could be utilized for the calculations. The average and maximum values of the aneurysm wall were evaluated, and *Ansys CFD Post (Ansys*, *Inc*.*)* was used to visualize the parameters.

### Statistical analyses

*JMP version 10 (SAS Institute Inc*., *Cary*, *USA)* was used for statistical analysis. Student’s *t*-tests were used to assess the mean and standard deviation of continuous variables. Statistical significance was set at *p* < 0.05.

## Results

### Changes of hemodynamic parameters in MCA aneurysms before and after growth

The maximum diameter of the aneurysms increased from 4.85 ± 0.90 mm to 5.88 ± 0.84 mm (*p* = 0.023) after aneurysm growth. The height of the aneurysms also increased from 3.51 ± 1.02 mm to 4.57 ± 1.64 mm (*p* = 0.123). The time-averaged WSS and WSSG and blood flow velocity changed from 4.11 ± 3.23 Pa to 2.16 ± 1.97 Pa (*p* = 0.92), from 2444 ± 1937 Pa/mm to 1254 ± 834 Pa/mm (*p* = 0.33), and from 0.51 ± 0.17 mm/s to 0.39 ± 0.19 mm/s (*p* = 0.10), respectively, without statistical significance (Table 1). The maximum and average OSI tended to increase from 0.27 ± 0.11 to 0.34 ± 0.98 (*p* = 0.07) and from 0.0078 ± 0.0065 to 0.0114 ± 0.0113 (*p* = 0.20), respectively, after aneurysm growth. No significant changes were observed in the PD and wall pressure before and after aneurysm growth (Table 1).

**Table 1:** Hemodynamic parameter changes before and after enlargement of aneurysms.

Factor	Pre-enlargement	Post-enlargement	P value
**Max diameter (mm)**	4.85±0.90	5.88±0.84	0.023
**Height (mm)**	3.51±1.02	4.57±1.64	0.123
**Hemodynamic parameters**			
**Time-averaged-WSS (Pa)**	4.11±3.23	2.16±1.97	0.92
**Time-averaged-WSSG (Pa/mm)**	2444±1937	1254±834	0.33
**OSI average**	0.0078±0.0065	0.0114±0.0113	0.20
**OSI max**	0.27±0.11	0.34±0.98	0.07
**PD max**	0.88±0.28	0.82±0.19	0.71
**Velocity (m/s)**	0.51±0.17	0.39±0.19	0.10
**Wall pressure (Pa)**	1375±683	1305±895	0.57

MAX, maximum value; WSS, wall shear stress; WSSG, wall shear stress gradient; OSI, oscillatory shear index; PD, pressure difference

### Clinical course and hemodynamic changes of 9 MCA aneurysms with aneurysm growth

Fig 1 and Table 2 present the clinical course and hemodynamic changes in nine patients with MCA aneurysms before and after aneurysm growth. Seven of the nine aneurysms (78%) were Type 1, whereas Types 2 and 3 had one aneurysm each. Three of the seven (43%) Type 1 aneurysms ruptured after aneurysm growth, and four (57%) remained unruptured. Aneurysmal rupture developed within 1 year of the aneurysm growth diagnosis in all ruptured cases. In unruptured cases, more than 3 years had passed without aneurysmal rupture after the diagnosis of aneurysm growth.

**Table 2:** Clinical course and hemodynamic changes of 9 cases of enlarged MCA aneurysms.

Growth pattern	Case	Clinical course	Sex	Age	Follow-up period (month)		Initial maximal diameter	Enlarged diameter	OSI max		PD max	
					Initial to growth	Growth to rupture / growth to last FU	(mm)	(mm)	Initial	After growth	Initial	After growth
Type 1 Homothetic	1	ruptured	F	85	30	6	5.9	1	0.334	0.459	1.114	0.94
	2	ruptured	M	68	107	10	5.8	1.2	0.397	0.447	1.001	0.855
	3	ruptured	F	89	131	2	4.9	2.5	0.228	0.458	0.834	0.816
	average			81	89	6	5.5	1.6	0.320	0.455	0.984	0.87
	4	unruptured	F	74	86	50	5.5	1	0.055	0.316	1.395	1.148
	5	unruptured	F	68	67	37	4.7	1	0.193	0.307	0.539	0.753
	6	unruptured	M	66	36	84	3.2	2.2	0.286	0.377	0.627	0.773
	7	unruptured	M	77	6	42	4.4	2.2	0.391	0.276	0.63	0.424
	average			71	49	53	4.5	1.6	0.231	0.319	0.797	0.774
Type2 De novo bleb	8	ruptured	F	77	37	7	5.4	1.1	0.285	0.215	1.043	0.962
Type3 Bleb enlargement	9	unruptured	M	77	6	42	3.9	1.1	0.228	0.222	0.775	0.723

MAX, maximum value; OSI, oscillatory shear index; PD, pressure difference; FU, follow-up

In most Type 1 aneurysms (6/7, 86%), maximum OSI increased after growth regardless of ruptured or unruptured status (before vs. after [ruptured]: 0.32 ± 0.086 vs. 0.455 ± 0.007, *p* = 0.94, before vs. after [unruptured]:0.231 ± 0.142 vs. 0.319 ± 0.042, *p* = 0.84). The maximum OSI values after aneurysmal growth were significantly higher in ruptured than in unruptured Type 1 aneurysms (ruptured vs. unruptured: 0.455 ± 0.007 vs. 0.319 ± 0.042, *p* = 0.003). The values of maximum PD after growth did not differ between ruptured and unruptured Type 1 aneurysms (ruptured vs. unruptured: 0.870 ± 0.063 vs. 0.774 ± 0.296, *p* = 0.71). In Type 2 aneurysm (case 8), the maximum OSI decreased slightly from 0.285 to 0.215 despite subsequent IA rupture requiring clipping procedure.

### Representative cases

#### Type 1-ruptured

**Fig 3:**
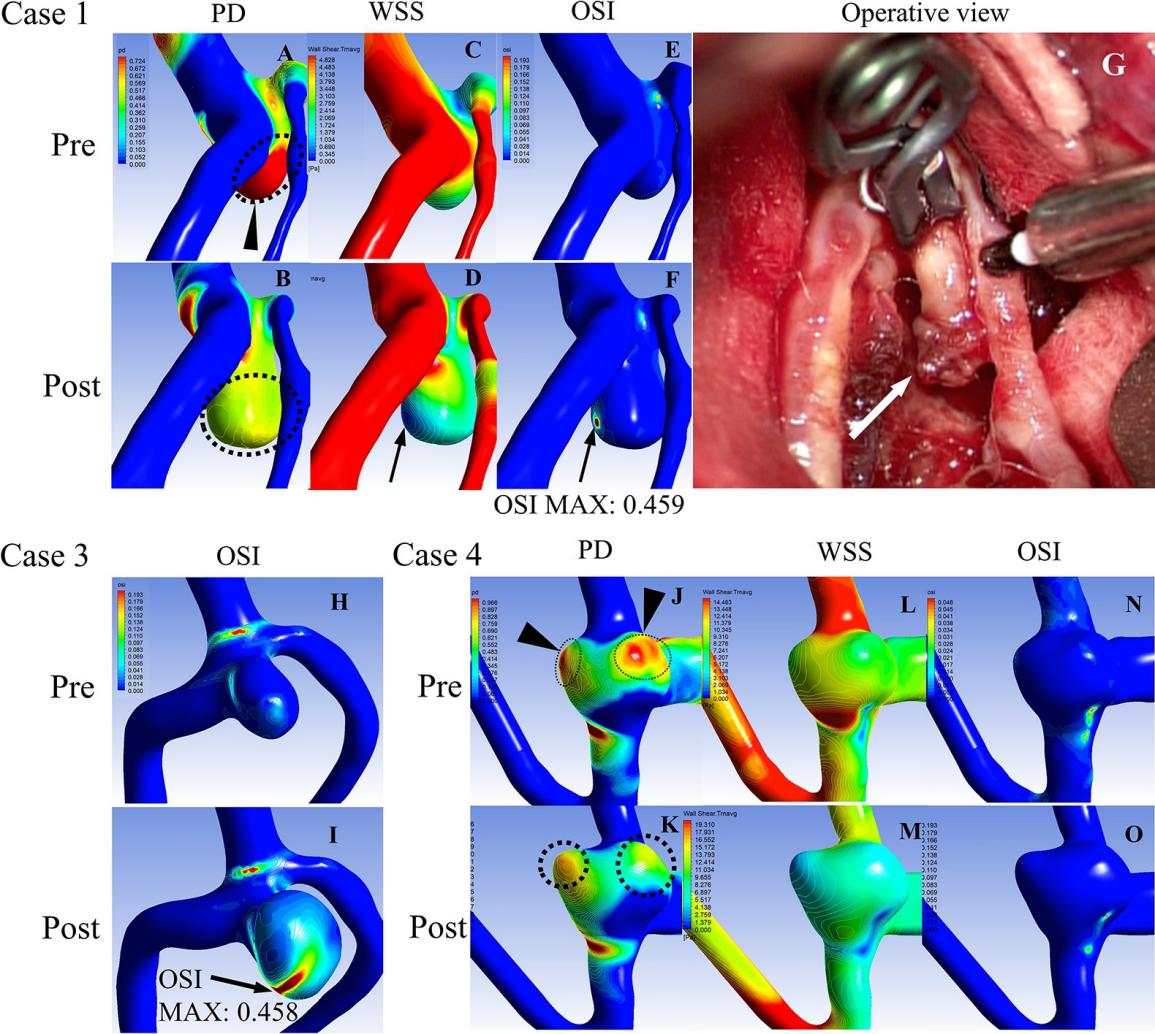
Hemodynamic changes of the representative cases in Type 1 aneurysms before and after aneurysm growth. **(A, B)** Distribution of the pressure difference (PD) before (A) and after aneurysm growth (B) in Case 1. A. The dotted circle with a black arrowhead indicates a high-PD area that corresponds to the direction of aneurysm growth. **(B)** The dotted circle indicates an enlarged aneurysmal sac. **(C, D)** Time-averaged wall shear stress (WSS) before (C) and after aneurysm growth (D) in Case 1. **(D)** The black arrow indicates the low-WSS region consistent with the presumed rupture point. **(E, F)** Oscillatory shear index (OSI) before (E) and after aneurysm growth (F) in Case 1. **(F)** The black arrow indicates the newly emerged focal high-OSI area, which corresponds to the low-WSS area and the presumed rupture point. **(G)** An intraoperative image of surgical clipping in Case 1. The white arrow indicates the rupture point of the thrombus without thinned aneurysm walls. **(H, I)** OSI before (H) and after aneurysm growth (I) in Case 3. The black arrow indicates a newly emerged spot with a high OSI. **(J, K)** Distribution of PD before (J) and after aneurysm growth (K) in Case 4. The dotted circle with a black arrowhead indicates a high-PD area that corresponds to the points of aneurysm growth. **(L, M)** Time-averaged WSS before (L) and after aneurysm growth (M) in Case 4. **(N, O)** OSI before (N) and after aneurysm growth (O) in Case 4.

Case 1 was a representative example of a ruptured Type 1 aneurysm. Before enlargement, a high-PD area was observed at the site where the blood flow impinged in a straight line from the parent vessel, and the aneurysm grew in the high-PD area (Fig 3A and 3B). Following enlargement, a localized area of high OSI with a surrounding low-WSS area (Fig 3C and 3D), which was not previously present, emerged in the aneurysm wall. Maximum OSI increased from 0.334 to 0.459 (Fig 3E and 3F). This newly emerged high-OSI area corresponded to the rupture point, as confirmed by the presence of a thrombus without thinned aneurysm wall during surgery (Fig 3G). Similarly, an increased OSI was observed in Cases 2 and 3 (Fig 3H and 3I). All the remaining type 1-ruptured cases are presented in S2 Fig. Case 2 had a severe SAH and was deemed unsuitable for surgery. Case 3 underwent endovascular treatment, making it challenging to confirm the rupture point.

#### Type 1-unruptured

Case 4 is representative of an unruptured Type 1 aneurysm. Aneurysm growth was observed in the high-PD area, similar to that in the ruptured cases (Fig 3J and 3K). Furthermore, a low WSS corresponded to a high PD area (Fig 3L and 3M). However, unlike the ruptured cases, no new high-OSI areas were observed (Fig 3N and 3O). All the remaining type 1-unruptured cases are presented in S2 Fig.

#### Type 2

**Fig 4:**
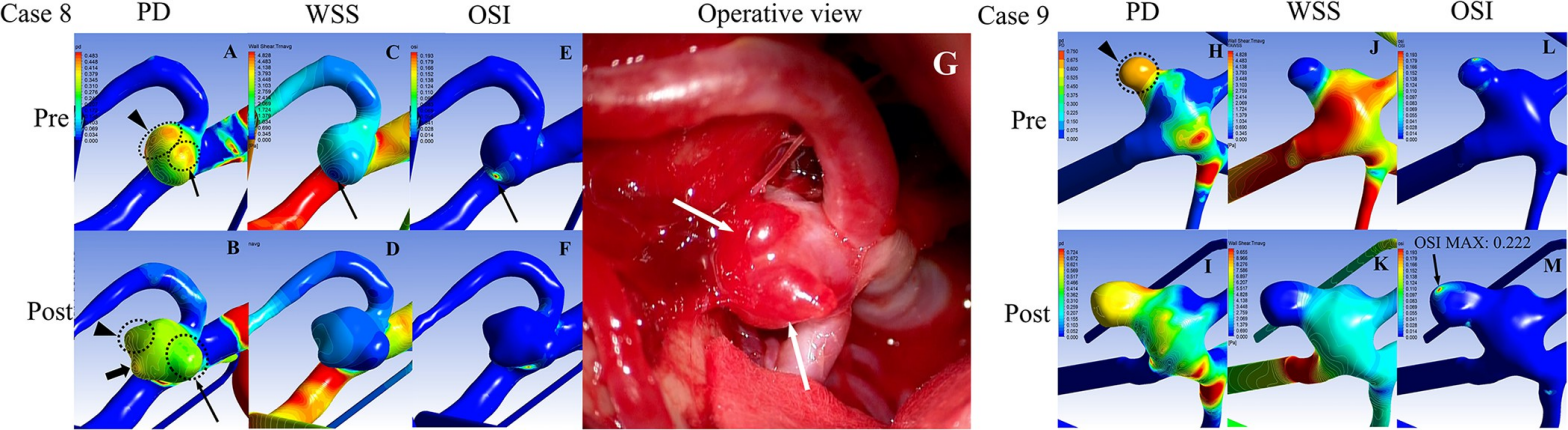
Hemodynamic changes of the representative cases in Types 2 and 3 aneurysms before and after aneurysm growth. **(A, B)** Distribution of pressure difference (PD) before (A) and after aneurysm growth (B) in Case 8. A. Dotted circles with the arrow and arrowhead indicate two high-PD areas that correspond to the direction of aneurysm growth. **(B)** Dotted circles indicate the enlarged aneurysmal sac. Dotted circles with the black arrow and arrowhead indicate high-PD areas before enlargement, which are consistent with newly formed blebs. Black thick arrow indicates another newly formed bleb consistent with the low WSS and high OSI areas before enlargement. **(C, D)** Time-averaged wall shear stress (WSS) before (C) and after aneurysm growth (D) in Case 8. **(C)** The black arrow indicates low-WSS areas. **(E, F)** Oscillatory shear index (OSI) before (E) and after aneurysm growth (F) in Case 8. (**E)** The black arrow indicates a high OSI, which corresponds to bleb formation. **(G)** An intraoperative image of surgical clipping in Case 8. White arrows indicate two blebs with thinning vessel walls, which correspond to the high-PD area in (B). **(H, I)** Distribution of PD before (H) and after aneurysm growth (I) in Case 9. **(H)** The dotted circle with a black arrowhead indicates the high-PD area that corresponds to the bleb. **(I)** The dotted circle indicates an enlarged bleb. **(J, K)** Time-averaged WSS before (J) and after aneurysm growth (K) in Case 9. **(M)** Hemodynamic images of the aneurysm after growth in Case 9. **(L, M)** OSI before (L) and after aneurysm growth (M) in Case 9. **(M)** Black arrow indicates a relatively high OSI.

Two regions of high PD were observed before the aneurysm growth, corresponding to the points of bleb formation after aneurysm growth (Case 8, Fig 4A and 4B, two blebs indicated by a dotted circle).). At the point of future bleb formation, a focal high OSI was observed with a surrounding low WSS before aneurysm growth (Fig 4B, 4C and 4E, indicated by a thick arrow). However, no specific findings were observed for WSS or OSI after growth (Fig 4D and 4F). During the clipping procedure performed just after the rupture, two blebs with thinned aneurysm wall consistent with high PD were found, however, it was unclear which one had ruptured (Fig 4G).

#### Type 3

Before bleb enlargement, the low WSS and high PD were limited to the bleb wall (Case 9, Fig 4J, 4K, 4M and 4H). Although a slight increase in OSI was observed in the bleb before growth, the maximum value did not increase after growth (before: 0.228, after: 0.222, Fig 4L and 4M).

## Discussion

In this study, we examined the hemodynamic features of nine MCA aneurysms before and after growth using CFD analysis. Three growth patterns were observed, and the hemodynamic changes differed depending on these patterns. In the homothetic growth group, the maximum OSI after aneurysm growth was significantly higher in ruptured cases than in unruptured cases. Furthermore, a newly emerged high-OSI area after aneurysm growth was associated with the rupture point. Unfortunately, only one case among the Type1 ruptured cases was confirmed under direct observation.

This is the first study to analyze the hemodynamic changes before and after aneurysmal growth and the differences between ruptured and unruptured cases.

Growth patterns in the present study were divided into homothetic growth (Type 1), de novo bleb formation (Type 2), and bleb enlargement (Type 3). A previous report classified enlarged small aneurysms into two groups: a focal growth group with bleb or blister formation and a global growth group with enlargement accompanied by neck broadening [12]. In the focal growth group, a high OSI and focal low WSS corresponded to the point of morphological change [12]. This hemodynamic feature was similar to that observed before aneurysm growth in Type 2 aneurysms in the present study. Moreover, the same hemodynamic features were observed after aneurysm growth at the rupture point in Type 1 cases. Several previous reports have indicated this notable feature of high OSI and low WSS [11, 13]. Using CFD analysis of 65 aneurysms, Cebral et al. revealed that a lower WSS and higher OSI were associated with atherosclerotic changes in the aneurysmal wall.

Due to the difficulty of obtaining serial images over time before and after the morphological changes, most CFD-related studies have focused on single-time-point assessments. However, a previous study examined morphological and hemodynamic changes during cerebral aneurysm growth using MRA at two time points similar to this study [14]. They found that geometry bulk growth occurred in areas of low WSS, and kinetic energy (KE) increased with correlation to the change in aneurysm volume from the first time point to the second. In the present study, although KE and the volume change were not assessed, aneurysm local growth in the low WSS region was observed in type 2. Furthermore, the high PD area was useful for predicting the growing area and direction of aneurysm growth rather than low WSS because all patients demonstrated aneurysm growth in the direction of high PD, irrespective of the growth pattern. On the other hand, in the case series of Nordah et al., a newly emerged high OSI area was observed in one case, like ruptured type 1 in our study, while in the other three cases, the maximum value of the high OSI area was reduced. However, the relationship with rupture is uncertain because they did not observe the clinical events after enlargement. Another study reported a case of multiple aneurysms that ruptured following growth, analyzing CFD using three serial images: the initial image, the time of growth, and the time of rupture [15]. In the ruptured MCA aneurysm, they observed the appearance of low WSS that corresponded to the enlarged region and an increase in OSI at the ruptured area. Furthermore, they reported that the average OSI within the aneurysm sac increased over time. Although we focus primarily on the maximum OSI value in our study, these findings indicate that low WSS and OSI changes are potentially correlated with the enlargement of aneurysms and the incidence of subsequent rupture.

From a pathological perspective, a previous report demonstrated macrophage infiltration in the growing regions of aneurysms exposed to low WSS and high OSI in a rat model [16]. Similarly, low WSS and high OSI upregulate endothelial surface adhesion molecules, leading to flow-induced nitric oxide dysfunction, increased endothelial permeability, and inflammatory cell infiltration in other basic studies [17, 18]. Inflammatory changes triggered by high OSI and low WSS may cause instability of the aneurysmal wall, leading to morphological changes in aneurysmal walls, including aneurysm growth, wall thickness variations, and aneurysmal rupture. However, the extremely high OSI and low WSS patterns detected at the rupture point in Type 1 aneurysms were not observed in the bleb wall of the Type 2 case. The underlying mechanism for this discrepancy is unclear in our study with limited cases; however, the rupture mechanism may differ between the homothetic enlargement pattern and the bleb formation case. In other words, this suggests that the mechanism of bleb wall rupture and the rupture of walls other than the bleb could be different. In previous reports [19–21], CFD analyses using aneurysms with bleb formation have highlighted that the hemodynamic environment of the bleb walls is different from that of non-bleb walls, which could cause a bleb wall specific condition such as thinning. Intraoperative findings in our cases revealed thinning of the aneurysm wall localized to the bleb in Case 8 (Type 2: de novo bleb formation), while no obvious thinning was observed at the ruptured IA wall in Case 1 (Type 1: homothetic enlargement). Unfortunately, intraoperative findings were only confirmed in these two cases, the mechanism of rupture of thickened aneurysm walls and thinned aneurysm walls (corresponding to the bleb wall) is potentially different. Meng et al. summarized the relationship between thin-wall changes, thick-wall changes, and rupture, including the relationship with OSI and WSS [22]. They suggested that low WSS combined with high OSI is related to atherosclerotic changes and inflammatory infiltration, while high WSS combined with positive WSSG is associated with mural cells producing matrix metalloproteinases (MMPs) and possible bleb, and each pathway may contribute to IA rupture. This high and low WSS theory implies that the more than two mechanisms of IA rupture may exist. This study also supports the idea that the mechanisms for rupture at the bleb site and rupture in the walls other than the bleb are different.

The present study had several limitations. First, the case series was derived from a single center, and the number of cases included was too small to obtain sufficiently meaningful predictive value. In particular, there were only a few cases of Types 2 and 3 in our entire database, and they often required early treatment, leading to limited opportunities to obtain data. Therefore, further research on these two subtypes is necessary.

Second, all images used to obtain the morphological data of the aneurysms and parent arteries were obtained using MRA; using CTA or DSA with a high resolution is desirable for accurate CFD analysis. The growth pattern classification was also evaluated based on comparisons made with the estimated shape after enlargement. Although it was an objective evaluation, there is a possibility that this geometric classification differs from the actual morphological change, and the classifications might overlap among three types and might not be completely delineated.

Finally, patient-specific flow-velocity measurements were not performed. Instead, inlet flow velocity measurements from the literature were used as the input conditions. This is a common inflow condition from volunteers (mostly male) and may be physiologically different from this case series.

Despite these limitations, a CFD analysis comparing the morphological changes before and after aneurysm growth is novel and worthy of reporting.

## Conclusions

High-PD areas before aneurysm growth may predict the direction of aneurysm growth. High maximum OSI values after enlargement, particularly in cases with homothetic growth patterns, were potential risk factors for rupture. Further research with a larger sample size is necessary to validate these findings and elucidate the intricate mechanisms underlying aneurysm growth and rupture.

## Supporting information

S1 Fig Other cases of a ruptured Type 1 aneurysm (case2, 3). (PDF)

S2 Fig Other cases of a unruptured Type 1 aneurysm (case5-7). (PDF)

S1 Dataset Quantitative data for all cases analyzed in this study. (XLSX)
