# Supplementary material for: Hemodynamic differences determining rupture and non-rupture in middle cerebral aneurysms after growth
Source: PLoS One. 2024 Aug 22;19(8):e0307495. doi: 10.1371/journal.pone.0307495 (PMC11340937; doi:10.1371/journal.pone.0307495)
Supplement: S1 Fig — (PDF) [file pone.0307495.s001.pdf]

Supplementary figure. 1

Other cases of a ruptured Type 1 aneurysm (case2, 3)

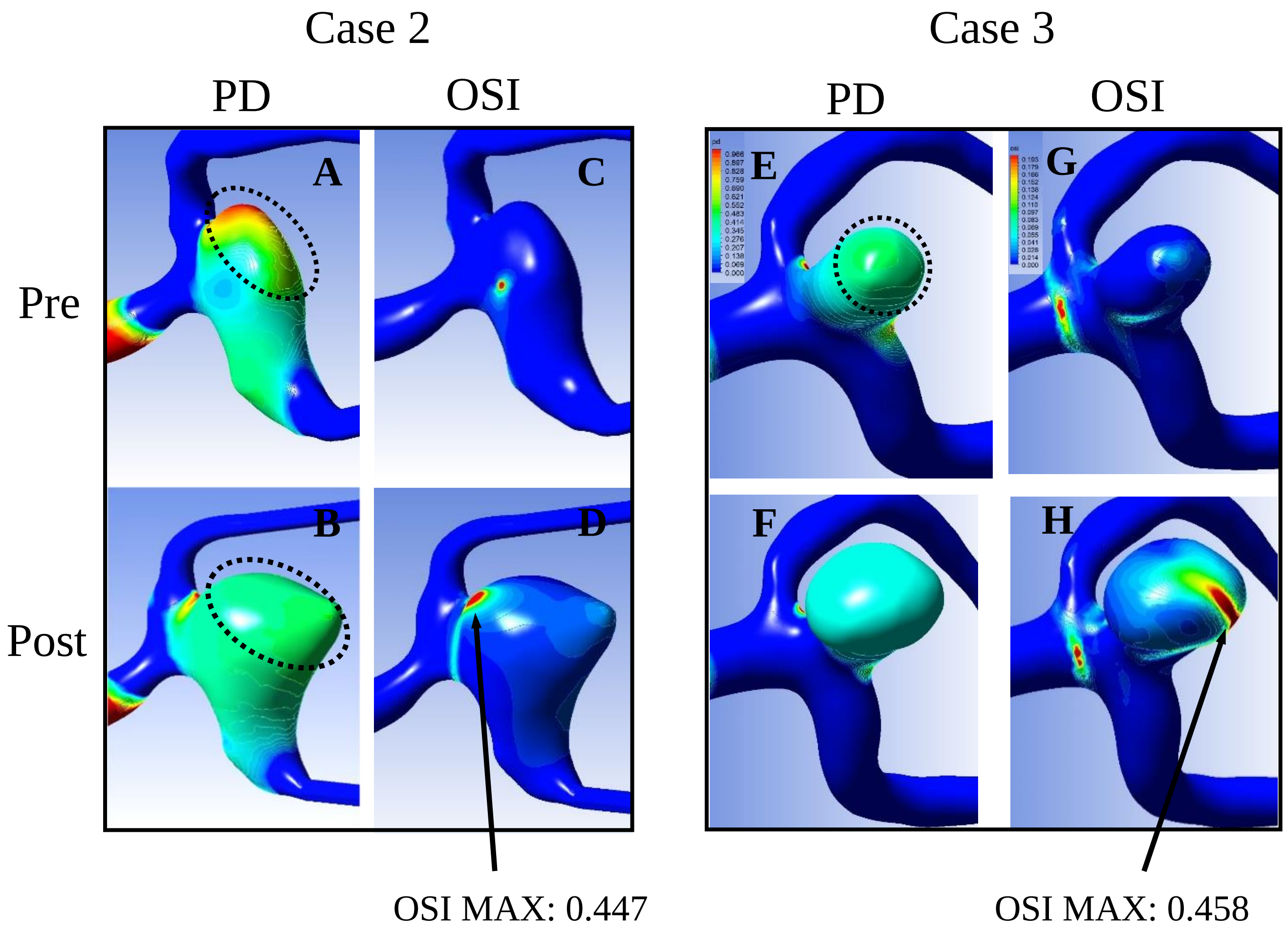

A, B. Distribution of the pressure difference (PD) before **(A)** and after aneurysm growth **(B)** in Case 2.

A. The dotted circle indicates a high-PD area that corresponds to the direction of aneurysm growth.

B. The dotted circle indicates an enlarged aneurysmal sac.

C, D. Oscillatory shear index (OSI) before **(C)** and after aneurysm growth **(D)** in Case 2.

D. The black arrow indicates the newly emerged focal high-OSI area. The maximum value of OSI is 0.447.

E, F. Distribution of the pressure difference (PD) before **(E)** and after aneurysm growth **(F)** in Case 3.

E. The dotted circle indicates a slightly high-PD area that corresponds to the direction of aneurysm growth.

G, H. Oscillatory shear index (OSI) before **(G)** and after aneurysm growth **(H)** in Case 3.

D. The black arrow indicates the newly emerged focal high-OSI area. The maximum value of OSI is 0.458.
