# Supplementary material for: Hemodynamic differences determining rupture and non-rupture in middle cerebral aneurysms after growth
Source: PLoS One. 2024 Aug 22;19(8):e0307495. doi: 10.1371/journal.pone.0307495 (PMC11340937; doi:10.1371/journal.pone.0307495)
Supplement: S2 Fig — (PDF) [file pone.0307495.s002.pdf]

Supplementary figure. 2

Other cases of a unruptured Type 1 aneurysm (case5-7)

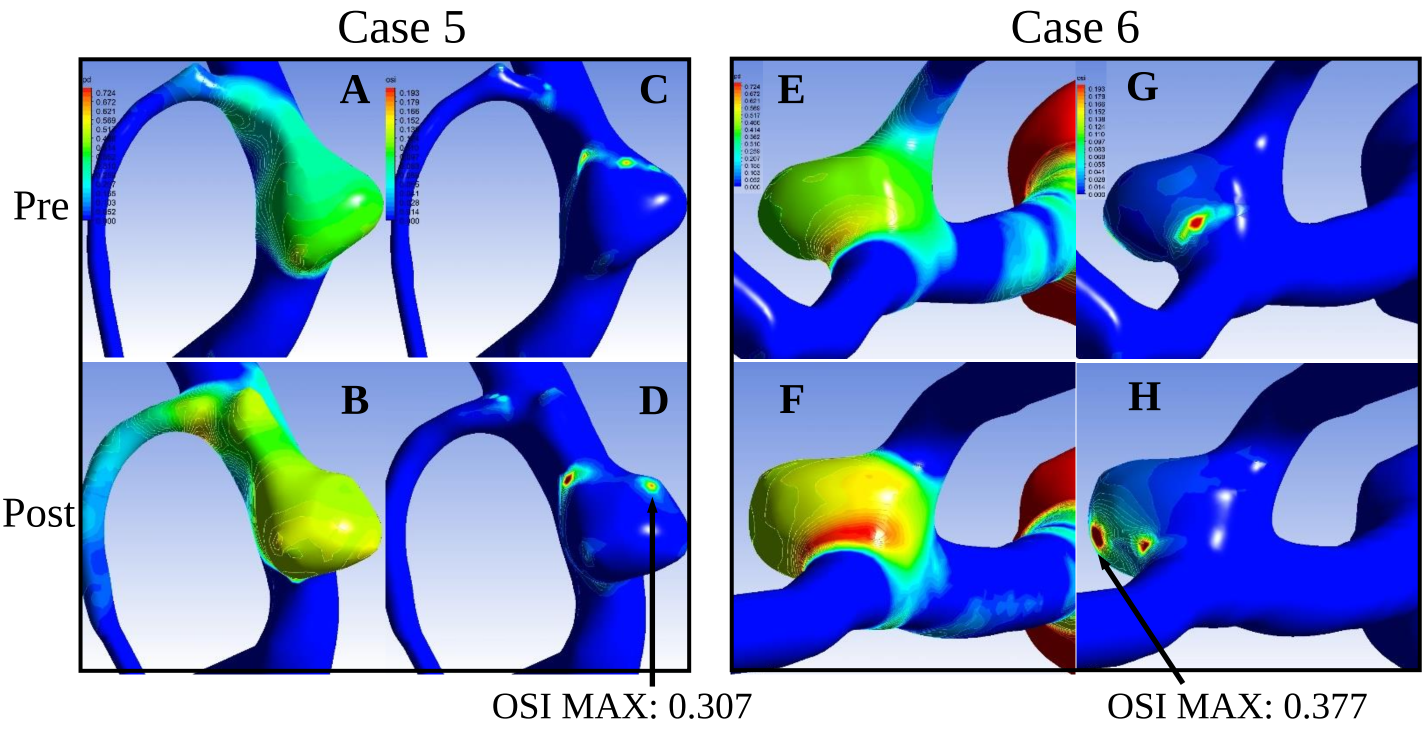

A, B. Distribution of pressure difference (PD) before **(A)** and after aneurysm growth **(B)** in Case 5.

C, D. Oscillatory shear index (OSI) before **(C)** and after aneurysm growth **(D)** in Case 5.

D. The black arrow indicates the newly emerged focal high-OSI area, but its maximum value is not so high.

E, F. Distribution of pressure difference (PD) before **(E)** and after aneurysm growth **(F)** in Case 6.

G, H. Oscillatory shear index (OSI) before **(G)** and after aneurysm growth **(H)** in Case 6.

H. The black arrow indicates the newly emerged focal high-OSI area, but its maximum value is not so high.

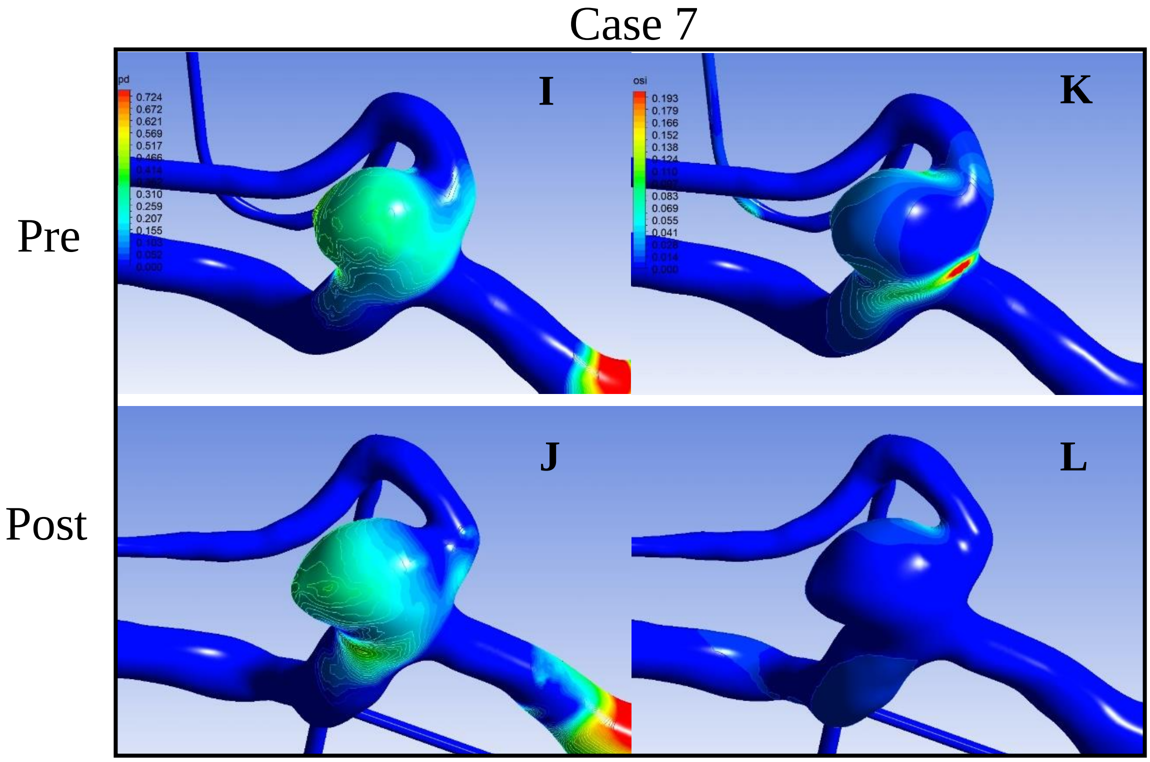

I, H. Distribution of pressure difference (PD) before **(I)** and after aneurysm growth **(H)** in Case 7.

K, L. Oscillatory shear index (OSI) before **(K)** and after aneurysm growth **(L)**. There is no newly high OSI area after growth.
